# Racial Differences in Prevalence and Clinical Characteristics of Asthma–Chronic Obstructive Pulmonary Disease Overlap

**DOI:** 10.3389/fmed.2021.780438

**Published:** 2021-11-22

**Authors:** Yong Suk Jo, Yong Il Hwang, Kwang Ha Yoo, Myung Goo Lee, Ki Suck Jung, Kyeong-Cheol Shin, Hyoung Kyu Yoon, Deog Kyeom Kim, Sang Yeub Lee, Chin Kook Rhee

**Affiliations:** ^1^Division of Pulmonary, Allergy, and Critical Care Medicine, Department of Internal Medicine, Hallym University Kangdong Sacred Heart Hospital, Seoul, South Korea; ^2^Division of Pulmonary, Allergy and Critical Care Medicine, Department of Internal Medicine, Hallym University Sacred Heart Hospital, Hallym University College of Medicine, Anyang, South Korea; ^3^Division of Pulmonary, Allergy and Critical Care Medicine, Department of Internal Medicine, Konkuk University School of Medicine, Seoul, South Korea; ^4^Division of Pulmonary, Allergy and Critical Care Medicine, Department of Internal Medicine, Hallym University Chuncheon Sacred Heart Hospital, Hallym University College of Medicine, Chuncheon, South Korea; ^5^Regional Center for Respiratory Disease, Yeungnam University Medical Center, Yeungnam University College of Medicine, Daegu, South Korea; ^6^Division of Pulmonary and Critical Care Medicine, Department of Internal Medicine, Yeouido St. Mary's Hospital, College of Medicine, The Catholic University of Korea, Seoul, South Korea; ^7^Division of Pulmonary and Critical Care Medicine, Department of Internal Medicine, Seoul Metropolitan Government-Seoul National University Boramae Medical Center, Seoul National University College of Medicine, Seoul, South Korea; ^8^Division of Pulmonary and Allergy Medicine, Department of Internal Medicine, Korea University Anam Hospital, Korea University, Seoul, South Korea; ^9^Division of Pulmonary and Critical Care Medicine, Department of Internal Medicine, Seoul St. Mary's Hospital, College of Medicine, The Catholic University of Korea, Seoul, South Korea

**Keywords:** ACO, asthma–COPD overlap, COPDGene (UA Genetic Epidemiology of COPD), KOCOSS (Korean COPD Subgroup Study), racial and ethnic differences, inhaled corticosteroid (ICS), exacerbation

## Abstract

**Background:** This study examined the differences in the prevalence and clinical features of asthma–chronic obstructive pulmonary disease (COPD) overlap (ACO) with identical diagnostic criteria by race and ethnicity in two nationwide cohorts of COPD.

**Methods:** We used data from the Korean COPD Subgroup Study (KOCOSS) and phase I of the US Genetic Epidemiology of COPD (COPDGene) study. We defined ACO by satisfying bronchodilator response (BDR) >15% and 400 ml and/or blood eosinophil count ≥300/μl.

**Results:** The prevalences of ACO according to ethnicity were non-Hispanic white (NHW), 21.4%; African American (AA), 17.4%; and Asian, 23.8%. Asian patients with ACO were older, predominantly male, with fewer symptoms, more severe airflow limitation, and fewer comorbidities than NHW and AA patients. During 1-year follow-up, exacerbations occurred in 28.2, 22.0, and 48.4% of NHW, AA, and Asian patients with ACO, respectively. Compared to patients with non-ACO from the same racial group, the risk for exacerbation was significantly higher in NHW and Asian patients with ACO [adjusted incident rate ratio (aIRR), 1.17; 95% CI, 1.01–1.36, and aIRR, 1.37; 95% CI, 1.09–1.71 for NHW and Asian patients with ACO, respectively]. Inhaled corticosteroid (ICS) reduced the risk for future exacerbation in total patients with ACO but the effect was not significant in each racial group.

**Conclusions:** The prevalence of ACO was similar in the two cohorts using the same diagnostic criteria. The risk for future exacerbation was significantly higher in ACO, and the use of ICS reduced the risk for exacerbation in total patients with ACO.

## Introduction

Asthma–chronic obstructive pulmonary disease (COPD) overlap (ACO) is a condition characterized by overlapping clinical features of asthma and COPD. It has recently been recognized as a disease, but debate remains whether it is a single disease entity distinct from asthma and COPD.

Patients with features of both asthma and COPD have been excluded from clinical studies on either disease, resulting in a lack of information about ACO from prevalence to course and outcomes of the disease. Furthermore, as there are as yet no unified diagnostic criteria for ACO, the exact prevalence of ACO is unknown because it varies according to the diagnostic criteria used to define the disease in each study with estimates ranging from between 9 and 55% (1).

Efforts have been made to identify patients with ACO in several chronic airway disease cohorts focused on either asthma or COPD. Several cohort studies have reported relatively consistent data on ACO, indicating that they have more severe respiratory symptoms, poorer quality of life status and lung function, experience more frequent exacerbations, and have higher mortality rates than patients with COPD alone (2–6). This clinical pattern of patients with ACO was similar in a Korean COPD cohort even defined using several different diagnostic criteria (7). Although there have been racial comparisons within a single cohort, no studies have compared racial differences in ACO between distinct large cohorts.

Therefore, this study was performed to evaluate differences in the prevalence of ACO using identical diagnostic criteria according to race and ethnicity in two large-scale nationwide cohorts of COPD and to compare the clinical parameters and the risk for exacerbation between patients with ACO of different races.

## Materials

### Study Population

We used data from the Korean COPD Subgroup Study (KOCOSS) cohort (2012–2019) and phase I (2008–2011) of the US Genetic Epidemiology of COPD (COPDGene) study. The KOCOSS is an ongoing, prospective multicenter observational cohort, which has recruited patients with COPD from 48 referral hospitals in the Republic of Korea (8). The inclusion criteria for the cohort were age ≥40 years and positive diagnosis of COPD by a pulmonologist based on respiratory symptoms and spirometry-confirmed fixed airflow limitation [post-bronchodilator forced expiratory volume in 1 s/forced vital capacity (FEV_1_/FVC) <0.70]. The COPDGene study is a prospective, observational, multicenter cohort that has over 10,000 non-Hispanic white (NHW) and African American (AA) subjects aged ≥45 years with a smoking history of at least 10 pack-years, with and without COPD (9, 10).

### Clinical Data

Detailed sociodemographic information, such as age, sex, smoking history, and education level, was collected in both KOCOSS and COPDGene cohorts. Data on symptoms of cough >3 months, phlegm >3 months, severity of dyspnea on the modified Medical Research Council Dyspnea Scale (mMRC), and on quality of life status through the COPD assessment test (CAT) and the St George's Respiratory Questionnaire (SGRQ) were included in the analysis. Acute exacerbation in the 12 months prior to enrollment, exercise capacity represented by 6-minute walking distance (6MWD), pulmonary function test results, and inhaler status and type prescribed were extracted at enrollment. Data on comorbidities, such as cardiovascular disease and asthma, were also collected.

### Definition of ACO

Only patients with COPD aged 45 years or older with a smoking history of at least 10 pack-years and post-bronchodilator FEV_1_/FVC <0.7 were included in this study. The criteria for ACO, i.e., bronchodilator response (BDR) >15% and 400 ml and/or blood eosinophil count ≥300/μl, were applied equally to both cohorts according to the updated Spanish Guidelines on the Management of Asthma (GEMA) (11).

### Subsequent Exacerbations

In the KOCOSS cohort, patients were followed up at least every 6 months, and moderate and severe exacerbation data were recorded at each visit. Moderate exacerbation was defined as leading to a visit to an outpatient clinic earlier than scheduled with a prescription for systemic steroids and/or antibiotics, whereas severe exacerbation was defined as leading to a visit to the emergency department or hospitalization. In the COPDGene cohort, patients were contacted every 3–6 months by automated telephone contact, web-based questions, and coordinator telephone calls. Exacerbation was defined as acute respiratory symptoms that required the use of either antibiotics or systemic steroids, and severe exacerbations were defined by the need for hospitalization (10, 12). The same definitions of exacerbations were applied in both cohorts.

### Statistical Analysis

We compared clinical features between patients with and without a diagnosis of ACO according to three racial and ethnic groups: NHW, AA, and Asian. Continuous and categorical variables were compared among the three groups by ANOVA and the chi-square test, respectively.

The risk for exacerbation during the 1-year follow-up period is difficult to assume equidispersion, thus it was analyzed using a negative binomial regression model, and various clinical factors, such as age, sex, smoking history in pack-years, FEV_1_ at baseline, total CAT score, mMRC dyspnea scale, and history of exacerbation prior to enrollment, were used in the multivariable analysis as covariates. The effects of inhaled corticosteroid (ICS) containing inhaler therapy at the baseline on exacerbation were subsequently analyzed.

All tests were two-sided, and *P* < 0.05 was taken to indicate statistical significance. All analyses were performed using STATA software (ver. 16; StataCorp, College Station, TX, USA).

## Results

### Study Subjects

**Figure 1:**
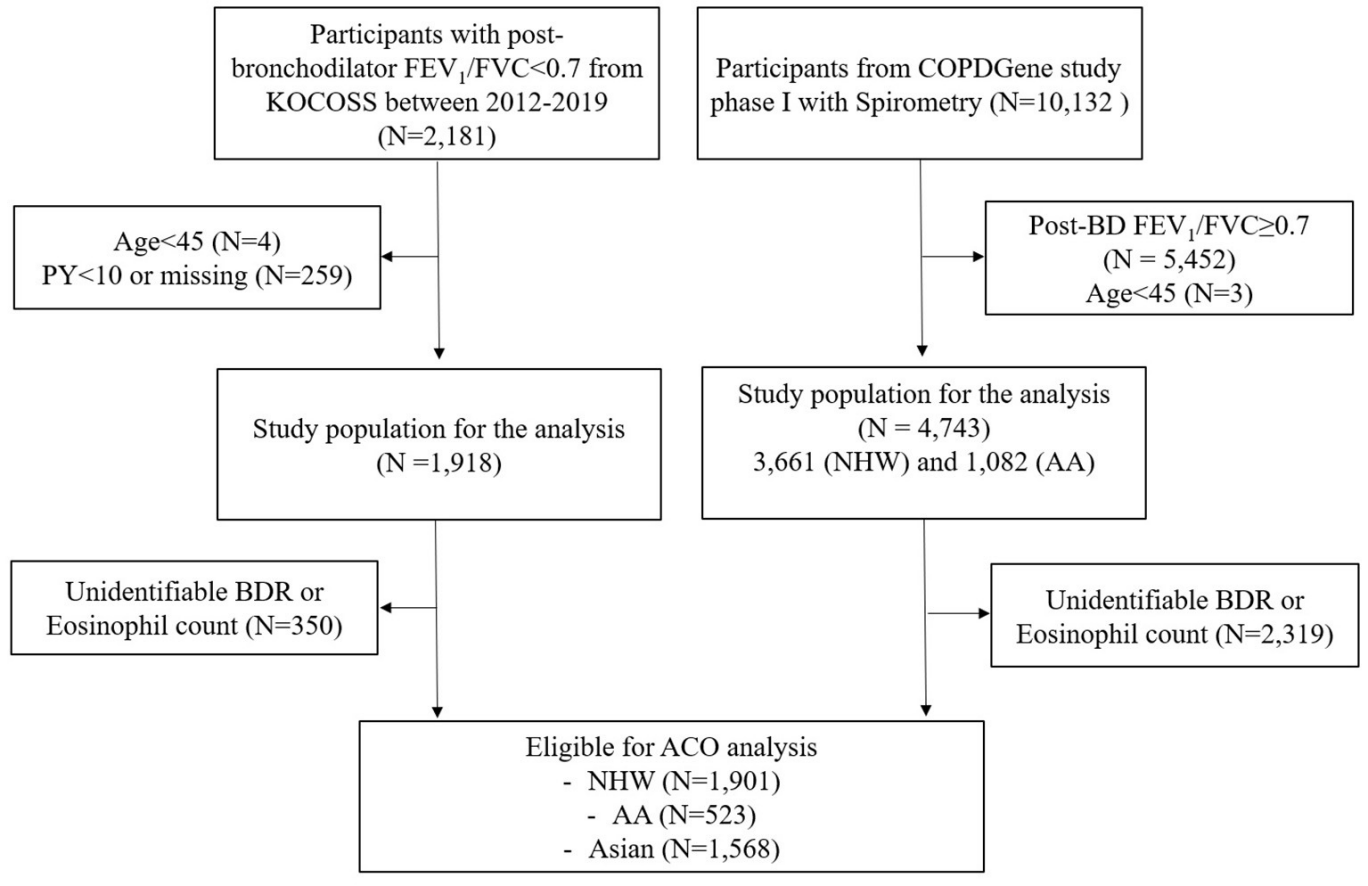
Flow diagram of the subjects in two cohorts. AA, African American; ACO, asthma–chronic obstructive pulmonary disease (COPD) overlap; BDR, bronchodilator response; COPD, chronic obstructive pulmonary disease; FEV_1_, forced expiratory volume in 1 s; FVC, forced vital capacity; KOCOSS, The Korean COPD Subgroup Study; NHW, non-Hispanic white; PY, pack-years.

The flows of study participants in each cohort are shown in Figure 1. A total of 2,181 COPD patients of KOCOSS from January 2012 to December 2019 were enrolled, and 1,568 COPD patients were eligible for analysis according to the ACO study design. Among these patients, 23.8% had ACO and 76.2% had non-ACO COPD.

In the COPDGene cohort, 10,132 subjects were enrolled at phase I, and 2,424 subjects were eligible for ACO analysis; 20.5% had ACO and 79.5% had non-ACO COPD. This patient group was divided into NHW and AA according to race. Among the NHW and AA patients, 21.4 and 17.4% were identified as having ACO, respectively (see Supplementary Figure 1). The prevalence of ACO was similar in the two cohorts but significantly lower in AA patients compared to NHW and Asian patients (*P* = 0.016).

### Clinical Features of ACO in the Three Racial Groups

Of the total 871 patients with ACO, 749 (86.0%) were identified by the blood eosinophil count ≥300/μl, 91 (10.7%) were identified by the extreme BDR criteria (>15% and 400 ml), and 29 (3.3%) met both criteria. The baseline characteristics of NHW (*n* = 407), AA (*n* = 91), and Asian (*n* = 373) patients with ACO are presented in Table 1. Asian patients with ACO were older, showed male predominance, and had lower body mass index (BMI) compared to those of other races. The Asian ACO patients had less frequent chronic bronchitis-related symptoms, such as chronic cough or phlegm lasting >3 months, and were less dyspneic than those of other races. Disease-related quality of life status was not significantly different between patients with ACO of the three racial groups. The mean blood eosinophil count was higher, and the proportion of current smokers was lower in Asian than AA patients with ACO. Education level and smoking pack-years were higher in NHW patients with ACO than those of the other racial groups. With regard to comorbidities, Asian patients with ACO had lower rates of ischemic heart disease, dyslipidemia, and gastroesophageal reflux disease.

**Table 1:** Baseline characteristics of participants with ACO by race and ethnicity.

	**NHW**	**AA**	**Asian**	***P***
	**(*n* = 407)**	**(*n* = 91)**	**(*n* = 373)**	
Age, years	64.0 ± 8.1	58.6 ± 8.0	68.2 ± 7.7	<0.001
Sex, male	265 (65.1)	50 (55.0)	368 (98.7)	<0.001
**Smoking status**				<0.001
Current smoker	131 (32.2)	62 (68.1)	122 (32.8)	
Ex-smoker	276 (67.8)	29 (31.9)	250 (67.2)	
Smoking, pack-years	50.4 ± 26.5	41.6 ± 22.4	46.5 ± 25.0	0.005
BMI (kg/m^2^)	28.7 ± 5.5	28.8 ± 6.5	23.1 ± 3.5	<0.001
Education (above high school)	167 (41.0)	13 (14.3)	63 (17.0)	<0.001
**Symptoms**				
Cough>3 months (*n* = 383)	176 (84.6)	32 (68.1)	46 (35.9)	<0.001
Phlegm>3 months (*n* = 378)	151 (83.4)	31 (72.1)	71 (46.4)	<0.001
mMRC	1.49 ± 1.38	1.77 ± 1.49	1.34 ± 0.93	0.007
CAT score	14.4 ± 8.2	16.6 ± 9.3	14.5 ± 8.0	0.066
**SGRQ score**				
Symptoms	38.1 ± 24.8	39.4 ± 26.0	42.7 ± 22.1	0.023
Impacts	21.3 ± 19.6	24.7 ± 23.7	25.1 ± 24.0	0.046
Activity	42.1 ± 27.9	46.5 ± 30.8	43.4 ± 28.9	0.422
Total	30.3 ± 21.2	33.7 ± 24.5	33.4 ± 22.1	0.108
Blood eosinophil count	405.5 ± 205.9	360.9 ± 162.0	526.5 ± 374.4	<0.001
Past exacerbation	145 (35.6)	31 (34.1)	85 (22.8)	0.001
Past severe exacerbation	55 (13.5)	23 (25.3)	39/362 (10.8)	0.001
**Comorbidities (*****n*** **=** **869)**				
*Cardiovascular disease*				
Hypertension	187 (46.0)	47 (51.7)	150 (40.4)	0.118
Congestive heart failure	16 (3.9)	3 (3.3)	12 (3.3)	0.863
Ischemic heart disease	71 (17.4)	8 (8.8)	14 (3.8)	<0.001
Peripheral vascular disease	19 (4.7)	1 (1.1)	7 (1.9)	0.060
Dyslipidemia	201 (49.4)	33 (36.3)	51 (13.7)	<0.001
Diabetes mellitus	52 (12.8)	14 (15.4)	59 (15.9)	0.444
Cerebrovascular disease (TIA, stroke) (*n* = 602)	16 (3.9)	3 (3.3)	3 (2.9)	0.862
Gastroesophageal reflux	141 (34.6)	18 (19.8)	50 (13.5)	<0.001
Past history of asthma (*n* = 814)	100 (27.9)	37 (43.0)	145 (39.2)	0.001

*Data are presented as the mean ± SD or number (%)*.

*AA, African American; ACO, asthma–COPD overlap; BMI, body mass index; CAT, COPD assessment test; COPD, chronic obstructive pulmonary disease; mMRC, modified Medical Research Council Dyspnea Scale; NHW, non-Hispanic white; SGRQ, St. George's Respiratory Questionnaire; TIA, transient ischemic attack*.

Asian patients with ACO showed the lowest FEV_1_ and FVC% predicted value and FEV_1_/FVC ratio, and the proportion of BDR positive patients was also lower compared to the other racial groups. Exercise capacity was higher in NHW patients with ACO (Table 2).

**Table 2:** Baseline pulmonary function test parameters of patients with ACO by race and ethnicity.

	**NHW**	**AA**	**Asian**	***P***
	**(*n* = 407)**	**(*n* = 91)**	**(*n* = 373)**	
Post-BD FEV_1_, L	1.97 ± 0.76	1.83 ± 0.72	1.75 ± 0.58	<0.001
Post-BD FEV_1_, %predicted	64.6 ± 20.8	67.4 ± 18.6	58.7 ± 18.4	<0.001
Post-BD FVC, L	3.48 ± 1.00	3.11 ± 1.01	3.40 ± 0.78	0.002
Post-BD FVC, %predicted	86.3 ± 18.0	90.1 ± 17.6	81.2 ± 16.4	<0.001
Post-BD FEV_1_/FVC	55.9 ± 11.7	58.4 ± 10.0	51.2 ± 11.9	<0.001
D_LCO_, %	70.3 ± 24.1	59.9 ± 20.9	64.7 ± 20.4	<0.001
BDR positivity^*^	136 (33.4)	33 (36.3)	64 (17.2)	<0.001
Exercise capacity, 6MWD (m)	419.4 ± 110.2	353.5 ± 115.3	378.5 ± 111.4	<0.001

*Data are presented as the mean ± SD or number (%)*.

* *Positive bronchodilator response was defined as a post-bronchodilator increase in FEV_1_ or FVC of at least 12% and 200 ml from baseline values at 15 minutes after inhalation of 400 μg salbutamol*.

*AA, African American; ACO, asthma–COPD overlap; BD, bronchodilator; COPD, chronic obstructive pulmonary disease; D_LCO_, diffusing capacity for carbon monoxide; FEV_1_, forced expiratory volume in 1 s; FVC, forced vital capacity; NHW, non-Hispanic white; 6MWD, 6-minute walking distance*.

**Figure 2:**
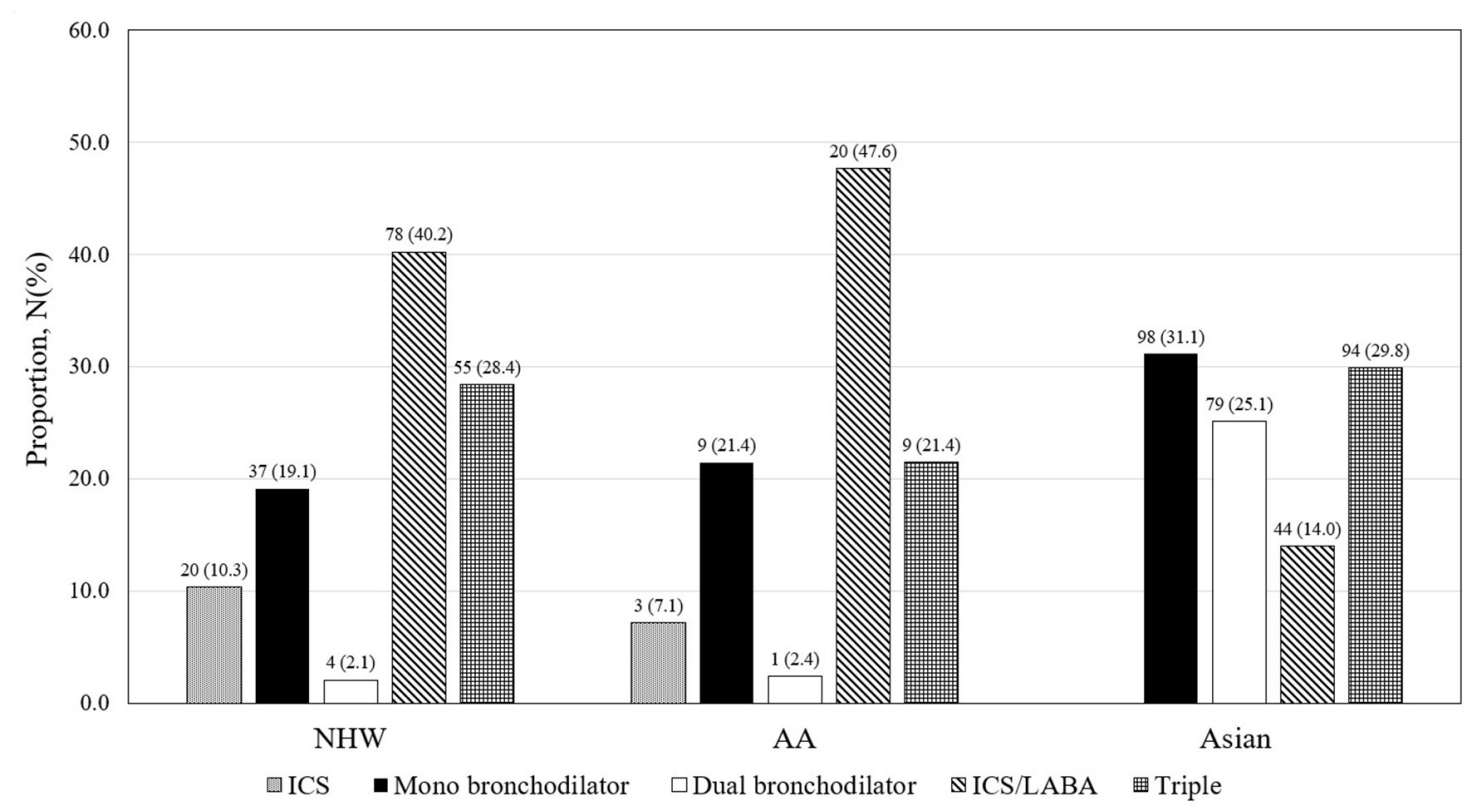
Inhaler prescription status in ACO of COPDGene and KOCOSS. AA, African American; ICS, inhaled corticosteroid; LABA, long-acting β_2_ receptor agonist; NHW, non-Hispanic white.

Inhaler treatment was prescribed in 47.7% (194 of 407) of NHW, 46.2% (42 of 91) of AA, and 84.5% (315 of 373) of Asian patients with ACO at baseline enrollment (Figure 2). In NHW and AA patients with ACO, ICS/long-acting β_2_ receptor agonist (LABA) combination inhaler was the most frequently used inhaler type, but mono-bronchodilator [either long-acting muscarinic antagonist (LAMA) or LABA] was the most frequently used in Asian patients. The proportion of patients who were prescribed any ICS, such as ICS/LABA and triple inhaler, was not significantly different among three racial groups of patients with ACO [37.6% (153 of 407) vs. 35.2% (32 of 91) vs. 37.0% (138 of 373) for NHW, AA, and Asian, respectively].

### Risk for Exacerbation and the Effects of ICS Treatment on Exacerbation Risk in Patients With ACO

During the 1-year follow-up, the risk for exacerbation was significantly higher in ACO patients than patients with non-ACO COPD (Table 3). Moderate-to-severe exacerbations were developed in 28.2% (53 of 188), 22.0% (9 of 41), and 48.4% (134 of 280) of NHW, AA, and Asian patients with ACO, respectively. Compared to patients with non-ACO COPD of the same racial group, the risk for moderate-to-severe exacerbation was significantly higher in NHW and Asian patients with ACO [adjusted incident rate ratio (aIRR) 1.17; 95% CI, 1.01–1.36; and aIRR 1.37; 95% CI, 1.09–1.71, respectively]. The rate of severe exacerbations was significantly higher in NHW patients with ACO than patients with non-ACO COPD (aIRR 1.31; 95% CI, 1.00–1.72).

**Table 3:** Risk for exacerbation during 1-year follow-up in patients with ACO according to race and ethnicity.

	**Case/*n***	**Frequency (mean ± SD)**	**Unadjusted IRR (95% CI)**	**Adjusted IRR (95% CI)**	
				**Model 1**	**Model 2**
**Moderate to severe AE**					
Total	196/506	0.86 ± 1.60	1.35 (1.19–1.53)^**^	1.28 (1.14–1.45)^**^	1.28 (1.14–1.45)^**^
NHW	53/188	0.56 ± 0.81	1.21 (1.04–1.41)^*^	1.21 (1.04–1.41)^*^	1.17 (1.01–1.36)^*^
AA	9/41	0.43 ± 0.68	1.03 (0.70–1.52)	1.05 (0.72–1.54)	1.05 (0.72–1.53)
Asian	134/280	1.43 ± 2.35	1.36 (1.06–1.74)^*^	1.36 (1.08–1.71)^*^	1.37 (1.09–1.71)^*^
**Severe AE**					
Total	51/465	0.18 ± 0.36	1.32 (1.10–1.59)^*^	1.27 (1.05–1.53)^*^	1.26 (1.04–1.52)^*^
NHW	13/166	0.21 ± 0.38	1.39 (1.07–1.82)^*^	1.35 (1.03–1.77)^*^	1.31 (1.00–1.72)^*^
AA	5/37	0.27 ± 0.38	0.82 (0.50–1.38)	0.86 (0.52–1.45)	0.86 (0.52–1.44)
Asian	36/280	0.27 ± 1.02	1.51 (0.96–2.36)	1.22 (0.79–1.88)	1.28 (0.83–1.98)

* *P < 0.05*,

** *P < 0.001*.

*Model 1 was adjusted for age, sex, smoking pack-years, BMI, mMRC, total CAT score, post-bronchodilator FEV_1_ (%predicted) at enrollment*.

*Model 2 was further adjusted for past exacerbation in the previous year (yes vs. no) in addition to Model 1*.

*IRR was calculated with reference to non-ACO*.

*AA, African American; ACO, asthma–COPD overlap; AE, acute exacerbation; BMI, body mass index; CI, confidence interval; COPD, chronic obstructive pulmonary disease; FEV_1_, forced expiratory volume in1 s; ICS, inhaled corticosteroid; IRR, incident rate ratio; NHW, non-Hispanic white; OR, odds ratio; PY, pack-years; SD, standard deviation*.

In total patients with ACO, the risk for moderate-to-severe exacerbation was significantly reduced in ICS users at the baseline compared to non-ICS users (aIRR 0.75; 95% CI, 0.57–0.98). However, the beneficial effects of ICS on exacerbation reduction were not significant in each racial group (Table 4).

**Table 4:** Effects of ICS on risk for exacerbation during 1-year follow-up in patients with ACO.

	**Case/*n***	**Frequency (mean ± SD)**	**Unadjusted IRR (95% CI)**	**Adjusted IRR (95% CI)**	
				**Model 1**	**Model 2**
**Moderate to severe AE**					
Total	196/506	0.85 ± 1.58	0.82 (0.63–1.07)	0.75 (0.57–0.98)^*^	0.75 (0.57–0.98)^*^
NHW	53/188	0.48 ± 0.72	1.02 (0.68–1.52)	0.84 (0.56–1.27)	0.81 (0.53–1.22)
AA	9/41	0.42 ± 0.84	2.03 (0.61–6.83)	2.31 (0.61–8.77)	2.34 (0.62–8.83)
Asian	134/277	1.16 ± 2.14	0.98 (0.64–1.51)	0.79 (0.53–1.19)	0.82 (0.55–1.24)
**Severe AE**					
Total	51/465	0.22 ± 0.68	1.13 (0.74–1.75)	0.89 (0.55–1.43)	0.87 (0.54–1.41)
NHW	13/166	0.14 ± 0.30	1.15 (0.55–2.38)	0.97 (0.45–2.08)	0.92 (0.43–2.00)
AA	5/37	0.24 ± 0.50	2.04 (0.35–12.0)	1.08 (0.16–7.36)	1.05 (0.15–7.16)
Asian	33/262	0.20 ± 0.73	1.16 (0.46–2.93)	0.68 (0.26–1.77)	0.68 (0.26–1.78)

* *P < 0.05*.

*Model 1 was adjusted for age, sex, smoking pack-years, BMI, mMRC, total CAT score, post-bronchodilator FEV_1_ (%predicted) at enrollment*.

*Model 2 was further adjusted for past exacerbation in the previous year (yes vs. no) in addition to Model 1*.

*IRR was calculated with reference to the non-ICS user in patients with ACO*.

*AA, African American; ACO, asthma–COPD overlap; AE, acute exacerbation; BMI, body mass index; CI, confidence interval; COPD, chronic obstructive pulmonary disease; FEV_1_, forced expiratory volume in1 s; ICS, inhaled corticosteroid; IRR, incident rate ratio; NHW, non-Hispanic white; OR, odds ratio; PY, pack-years; SD, standard deviation*.

In a separate analysis only of patients with ACO with blood eosinophil count ≥300/μl, ICS use at the baseline reduced the risk for moderate-to-severe exacerbation in total subjects, but the effect was not significant in each racial group (see Supplementary Table 1).

## Discussion

The prevalence rates of ACO using identical diagnostic criteria composed of BDR and blood eosinophil count were similar in two cohorts but lower in the AA group when divided into three racial and ethnic groups. Asian patients with ACO were more likely to be male, older, with lower BMI and smoking pack-years, and fewer symptoms (fewer dyspneic and chronic bronchitis symptoms) compared to the NHW and AA patients with ACO. Lung function was lower in Asian patients with ACO, and the proportion of BDR positive subjects was small, and they were prescribed more maintenance inhaler treatment. Patients with ACO of the three racial groups experienced moderate-to-severe exacerbations more frequently compared to patients with non-ACO, and although the effects were not significant within each racial group, the use of ICS in total patients with ACO reduced moderate-to-severe exacerbation risk.

We defined ACO by BDR >15% and 400 ml and/or blood eosinophil count ≥300/μl among patients with COPD classified by age, smoking history, and fixed airflow limitation. As there are no unified diagnostic criteria for ACO, studies have yielded inconsistent results depending on the criteria used. We applied the same criteria consistent with the most recently proposed definition of ACO by the Spanish Guidelines for COPD and GEMA (11, 13). The results indicated similar prevalence rates of ACO among the three racial groups.

Asian patients with ACO are more likely to be older, male, and have less smoking history compared to NHW and AA patients with ACO. The low percentage of females among Asian patients with ACO was due to the low rate of smoking among females in Asia. The results of this study were compatible with previous reports. A previous prospective study indicated that the percentage of females among Asian patients with COPD was 5% (14), and the percentage of females in the IMPACT trial in Asia was 5% (15). In addition, Asian patients with ACO had significantly lower BMI compared to NHW and AA patients, which may have been due to genetic factors and differences in nutrition and dietary habits. The low BMI in Asian patients with COPD was consistent with previous studies. For example, the mean BMI in the Hokkaido (Japanese) cohort was 22 kg/m^2^ compared to 27 kg/m^2^ in the ECLIPSE cohort (16), and the mean BMI of Asian subjects in the FLAME study was 22.4 kg/m^2^ (17).

With regard to comorbidities, compared to NHW and AA groups, Asian patients with ACO had lower rates of ischemic heart disease, dyslipidemia, cerebrovascular disease, and gastroesophageal reflux disease. Similar patterns were reported in previous COPD studies (18–20). As smoking is well-known to be the leading cause of COPD and atherosclerosis-related vascular disease (21), both environmental and genetic factors may have contributed to these findings. In this study, the prevalence of the previous history of asthma was significantly higher in AA and Asian patients with ACO compared to NHW patients. Compared to NHW, AA patients have a higher prevalence of childhood asthma (22), and this maybe related to the relatively younger age in the AA group in the present study, and Çolak et al. (23) reported that the percentage of patients with asthma was significantly elevated in cases of early COPD. These sociodemographic characteristics may interact with the prevalence of comorbidities as risk factors for COPD and ACO, and it is assumed that they also contributed to the observed racial differences. When the demographic and clinical features of patients with non-ACO COPD in the three racial and ethnic groups were compared, similar to those observed in patients with ACO were identified, although there were some differences (data are not shown).

There is broad agreement that patients with ACO experience frequent exacerbations, have a poor quality of life, more rapid decline in lung function, higher mortality rate, and greater use of healthcare resources compared to patients with asthma or COPD alone (1). We further compared patients with ACO and non-ACO in each cohort. In COPDGene, ACO subjects had a male predominance with fewer current smokers and higher rates of asthma history, favorable lung function, more BDR positivity, and higher blood eosinophil count than patients with non-ACO. Inhaler prescription status did not differ between ACO and non-ACO patients (see Supplementary Table 2). In KOCOSS, patients with ACO were also older, more BDR positivity, with higher blood eosinophil count compared to patients with non-ACO. There were no differences in previous asthma history or inhaler prescription status between patients with ACO and non-ACO (see Supplementary Table 3). These results were consistent with previously reported clinical findings in patients with ACO. During 1 year of follow-up, patients with ACO experience more exacerbations than non-ACO patients, and the difference was significant in NHW and Asian racial groups. Moreover, the rates of severe exacerbations were significantly higher in total and NHW patients with ACO. As the risk for exacerbations tends to be higher in patients with ACO than patients with non-ACO (6, 24, 25), our results were consistent with previous studies despite the low prescription rate of maintenance inhaler treatment regardless of ACO or non-ACO.

Although Asian patients with ACO had fewer past exacerbations than other ethnic group of ACOs, they experienced more exacerbations during 1-year follow-up. This is thought to be related to several risk factors of subsequent exacerbation risk, such as older age, had lower BMI, lower lung function, higher eosinophil count, and male predominance in Asian patients with ACO. Moreover, ~55.9% of patients with COPD in COPDGene and 16.6% of patients with COPD in KOCOSS did not use any long-acting bronchodilator and/or ICS. This implies undertreatment of patients with COPD regardless of ACO in real-world practice and suggests that more active treatment is needed in these patients. Also, Asian patients with ACO were receiving more maintenance inhaler treatment at the time of enrollment, and this suggests that they may already be more severe patients than other ethnic group of ACOs.

There have been inconsistent reports regarding the effects of ICS use in patients with ACO (7, 26–28). Recently, it was reported that ICS has reduced the risk for exacerbation in the KOCOSS cohort, but the protective effect of ICS was found only in ACO by the GINA/GOLD criteria among the five sets of diagnostic criteria for ACO (7). As asthma and COPD are umbrella labels for heterogeneous conditions, each of which includes several different clinical phenotypes with several different underlying mechanisms, ACO is also considered a clinical term including several different clinical phenotypes. Therefore, the use of ICS in ACO may or may not be protective in some cases, and this will depend mainly on the clinical features that determine the diagnosis of ACO. In this study, we found a protective effect of ICS in risk reduction of moderate-to-severe exacerbation in total patients with ACO, but the effect was not significant in any racial group of patients with ACO. On the other hand, the use of ICS in total patients with non-ACO led to increased risk for exacerbation (aIRR, 1.30; 95% CI, 1.11–1.54 for moderate-to-severe exacerbation and aIRR, 1.38; 95% CI, 1.05–1.83 for severe exacerbation), and the effects were also significant in Asian patients with non-ACO COPD. In fact, ICS was prescribed similarly in patients with non-ACO and ACO of all races (33.5 vs. 37.6% in NHW, 38.4 vs. 35.2% in AA, and 34.4 vs. 37.0% in Asian patients with non-ACO vs. ACO, respectively). This may have influenced the effects of ICS on exacerbation in both patients with ACO and non-ACO. There is some debate regarding the protective effect of ICS on exacerbation in ACO, but as it maybe harmful in patients with non-ACO, more attention will be needed to reduce overuse of ICS in COPD, particularly in patients with non-ACO.

This study had some limitations. First, KOCOSS is a single nationwide cohort study. Although Korean patients with COPD had similar overall clinical features to other Asian patients with COPD, such as Japanese, Chinese, and Taiwanese patients, the degree of exposure to cigarette smoke or biomass fuels differs between countries (18), which may limit the generalizability of our findings to patients with COPD in other Asian countries. Second, considering the GOLD recommendation of the “ABCD” assessment since 2011, the differences in the timing of patient enrollment in the two cohorts may have affected therapeutic approaches. Third, we used extremely high BDR to define ACO, which is similar to the previous study (29), but there were no quantitative chest CT data in the KOCOSS cohort, which made it impossible to compare differences in imaging findings, such as the proportion of emphysema, across the ACO groups. Fourth, another criterion for identifying ACO in our study was high blood eosinophil count. There has been a report that the risk of exacerbation increases in patients with COPD whose blood eosinophil counts of 300/μl or greater (30), but identifying high blood eosinophil count in COPD as a marker of eosinophilic COPD or ACO is controversial. Fifth, to apply the same criteria for defining COPD, non-smoking COPD could not be considered by including smoking history, thus, there is a possibility of bias due to the difference in smoking rates according to gender in the two countries. Sixth, only the use of ICS at the baseline was included in the analysis, so the number of cases in which inhaler therapy was changed during the follow-up period was not reflected. Lastly, exacerbations were ascertained differently in the American and Korean COPD cohorts. Korean subjects were interviewed in person every visit, thus, the frequency of exacerbations maybe higher in Korean subjects.

In conclusion, this is the first report of similar prevalence rates of ACO among three different race and ethnicity groups using identical diagnostic criteria in large COPD cohorts. There were several differences in clinical features and inhaler prescription patterns, but the tendency of increased risk for exacerbation in ACO was similar across all racial groups. The use of ICS in ACO reduced moderate-to-severe exacerbation risk during 1 year of follow-up, but the effect was not significant in each racial group of patients with ACO. However, the use of ICS in patients with non-ACO COPD increased the risk for exacerbation. Therefore, further attention and greater caution are needed with regard to the use of ICS in patients with COPD with a low likelihood of ACO.

## Data Availability Statement

The original contributions presented in the study are included in the article/Supplementary Material, further inquiries can be directed to the corresponding author.

## Ethics Statement

All hospitals involved in the KOCOSS and COPDGene cohort studies obtained approval from the relevant Institutional Review Board and patients provided informed consent, including at KONKUK University Medical Center (IRB No. KHH1010338). The patients/participants provided their written informed consent to participate in this study.

## Author Contributions

YSJ and CKR take responsibility for the data and analysis and designed the study. Members of the Korean Asthma Research Group contributed to the initiation of the ACO project and data acquisition. YSJ performed statistical analysis of data and wrote the first draft of the manuscript. All authors provided critical reviews. All authors contributed to the article and approved the submitted version.

## Funding

This research was supported by the Research Program funded Korea National Institute of Health (2016ER670100, 2016ER670101, 2016ER670102, 2018ER67100, 2018ER67101, 2018ER67102, and 202104044B8-00) for the KOCOSS cohort, and COPDGene was supported by Award Numbers U01 HL089897 and U01 HL089856 from the National Heart, Lung, and Blood Institute. COPDGene is also supported by the COPD Foundation through contributions made to an Industry Advisory Board that has included AstraZeneca, Bayer Pharmaceuticals, Boehringer-Ingelheim, Genentech, GlaxoSmithKline, Novartis, Pfizer, and Sunovion.

## Author Disclaimer

The content is solely the responsibility of the authors and does not necessarily represent the official views of the National Heart, Lung, and Blood Institute or the National Institutes of Health.

## Conflict of Interest

CR received consulting/lecture fees from MSD, AstraZeneca, GSK, Novartis, Takeda, Mundipharma, Boehringer-Ingelheim, Teva, Sanofi, and Bayer, but the sponsors had no role in the design of the study, the collection and analysis of data, or writing of the manuscript. The remaining authors declare that the research was conducted in the absence of any commercial or financial relationships that could be construed as a potential conflict of interest.

## Publisher's Note

All claims expressed in this article are solely those of the authors and do not necessarily represent those of their affiliated organizations, or those of the publisher, the editors and the reviewers. Any product that may be evaluated in this article, or claim that may be made by its manufacturer, is not guaranteed or endorsed by the publisher.

## Abbreviations

AA: African American
ACO: asthma–COPD overlap
ATS: American Thoracic Society
BDR: bronchodilator response
CAT: COPD assessment test
COPD: chronic obstructive pulmonary disease
FEV_1_: forced expiratory volume in 1 s
FVC: forced vital capacity
GINA: Global Initiative for Asthma
GOLD: Global Initiative for Chronic Obstructive Lung Disease
ICS: inhaled corticosteroid
IgE: immunoglobulin E
LABA: long acting beta2 receptor agonist
LAMA: long acting muscarinic receptor agonist
mMRC: modified Medical Research Council
NHW: non-Hispanic White
SGRQ: St. George's Respiratory Disease Questionnaire
6MWD: 6-minute walk distance.
