# Supplementary material for: Racial Differences in Prevalence and Clinical Characteristics of Asthma–Chronic Obstructive Pulmonary Disease Overlap
Source: Front Med (Lausanne). 2021 Nov 22;8:780438. doi: 10.3389/fmed.2021.780438 (PMC8645561; doi:10.3389/fmed.2021.780438)
Supplement: Supplementary file 1 [file Data_Sheet_1.docx]

**"Online Supplementary data"**

Yong Suk Jo and Chin Kook Rhee on behalf of the Korean Asthma Research Group & KOCOSS cohort

**Supplementary Table 1. Effects of ICS on exacerbation in patients with ACO by blood eosinophil count ≥300/μL**

|  | Case/*n* | Frequency (mean±SD) | Unadjusted IRR (95% CI) | Adjusted IRR (95% CI) | |
| --- | --- | --- | --- | --- | --- |
| Moderate to severe AE |  |  |  | Model 1 | Model 2 |
| Total | 183/459 | 0.92±1.67 | 0.83 (0.64-1.10) | 0.76 (0.57-1.00) | 0.75 (0.57-0.99)* |
| NHW | 45/158 | 0.58±0.82 | 0.99 (0.67-1.48) | 0.82 (0.54-1.24) | 0.80 (0.52-1.21) |
| AA | 7/33 | 0.45±0.73 | 2.84 (0.69-11.75) | 4.19 (0.86-20.49) | 4.08 (0.83-20.11) |
| Asian | 131/268 | 1.48±2.36 | 1.01(0.65-1.57) | 0.80 (0.53-1.21) | 0.83 (0.54-1.25) |
| Severe AE |  |  |  |  |  |
| Total | 54/441 | 0.23±0.71 | 1.18 (0.76-1.83) | 0.94 (0.58-1.53) | 0.93 (0.57-1.51) |
| NHW | 13/142 | 0.20±0.37 | 1.14 (0.55-2.38) | 0.97 (0.45-2.11) | 0.93 (0.43-2.02) |
| AA | 5/31 | 0.20±0.41 | 3.95 (0.34-45.32) | 3.08 (0.2-43.96) | 2.87 (0.19-43.84) |
| Asian | 36/268 | 0.28±1.04 | 1.21 (0.48-3.06) | 0.70 (0.27-1.83) | 0.70 (0.26-1.84) |

**P* <0.05

Model 1 was adjusted for age, sex, smoking pack-years, BMI, mMRC, total CAT score, postbronchodilator FEV1 (%predicted) at enrollment.

Model 2 was further adjusted for past exacerbation in the previous year (yes vs. no) in addition to Model 1.

IRR was calculated with reference to non-ACO patients.

AA, African American; ACO, asthma–COPD overlap; AE, acute exacerbation; BMI, body mass index; CI, confidence interval; COPD, chronic obstructive pulmonary disease; FEV_1_, forced expiratory volume in1 s; ICS, inhaled corticosteroid; IRR, incident rate ratio; NHW, non-Hispanic white; OR, odds ratio; PY, pack-years; SD, standard deviation.

**Supplementary Table 2. Baseline characteristics of patients with ACO and non-ACO in COPDGene**

**cohort**

|  | Non-ACO  (*n*=1,926 | ACO  (*n*=498) | *P*-value |
| --- | --- | --- | --- |
| Age, years | 62.6±8.3 | 63.0±8.3 | 0.250 |
| Sex, male | 990 (51.4) | 315 (63.3) | <0.001 |
| Smoking status, current smoker | 860 (44.7) | 193 (38.8) | 0.018 |
| Smoking, pack-years | 48.9±25.2 | 48.8±26.0 | 0.942 |
| BMI (kg/m^2^) | 28.1±5.9 | 28.7±5.7 | 0.055 |
| Education (above high school) | 691 (35.9) | 180 (36.1) | 0.912 |
| Symptoms |  |  |  |
| Cough >3months | 786 (83.9) | 208 (81.6) | 0.378 |
| Phlegm >3months | 661 (81.4) | 182 (81.3) | 0.958 |
| mMRC | 1.51±1.41 | 1.54±1.40 | 0.694 |
| CAT score | 14.2±8.6 | 14.8±8.5 | 0.165 |
| SGRQ score | 30.2±21.9 | 30.9±21.8 | 0.540 |
| Blood eosinophil count | 143.5±74.3 | 397.3±199.2 | <0.001 |
| Past exacerbation | 620 (32.2) | 176 (35.3) | 0.182 |
| Past severe exacerbation | 269 (14.0) | 78 (15.7) | 0.335 |
| Comorbidities |  |  |  |
| Cardiovascular disease |  |  |  |
| Hypertension | 862 (44.8) | 234 (47.0) | 0.377 |
| Congestive heart failure | 54 (2.8) | 19 (3.8) | 0.239 |
| Ischemic heart disease | 246 (12.8) | 79 (15.9) | 0.071 |
| Peripheral vascular disease | 58 (3.0) | 20 (4.0) | 0.258 |
| Dyslipidemia | 797 (41.4) | 234 (47.0) | 0.024 |
| Diabetes mellitus | 208 (10.8) | 66 (13.3) | 0.123 |
| Cerebrovascular disease (TIA, stroke) | 107 (5.6) | 19 (3.8) | 0.118 |
| Gastroesophageal reflux | 568 (29.5) | 159 (31.9) | 0.293 |
| Past history of asthma | 445 (25.6) | 137 (30.9) | 0.025 |
| Spirometry |  |  |  |
| Post-BD FEV_1_, L | 1.84±0.76 | 1.94±0.75 | 0.007 |
| Post-BD FEV_1_, %predicted | 64.0±21.3 | 65.1±20.4 | 0.304 |
| Post-BD FVC, L | 3.23±1.01 | 3.41±1.01 | <0.001 |
| Post-BD FVC, %predicted | 85.9±18.7 | 87.0±18.0 | 0.260 |
| Post-BD FEV_1_/FVC | 56.1±12.0 | 56.3±11.4 | 0.706 |
| D_LCO_, % | 66.1±23.0 | 68.6±23.9 | 0.045 |
| BDR positivity | 308 (16.0) | 169 (33.9) | <0.001 |
| Exercise capacity, 6MWD (m) | 411.9±112.3 | 407.2±114.0 | 0.416 |
| Medication | 833 | 236 |  |
| ICS | 45 (5.4) | 23 (9.8) |  |
| Mono bronchodilator | 145 (17.4) | 46 (19.5) | 0.006 |
| Dual bronchodilator | 21 (2.5) | 5 (2.1) |  |
| ICS/LABA | 299 (35.9) | 98 (45.5) |  |
| Triple | 323 (38.8) | 64 (27.1) |  |
| Any ICS | 667 (80.1) | 185 (78.4) | 0.571 |

Data are presented as the mean±standard deviation or number (%).

AA, African American; ACO, asthma–COPD overlap; BMI, body mass index; CAT, COPD assessment test; COPD, chronic obstructive pulmonary disease; D_LCO_, diffusing capacity for carbon monoxide; FEV_1_, forced expiratory volume in 1 s; FVC, forced vital capacity; ICS, inhaled corticosteroid; LABA, long-acting β_2_ receptor agonist; mMRC, Modified Medical Research Council Dyspnea Scale; NHW, non-Hispanic white; SGRQ, St. George’s Respiratory Questionnaire; TIA, transient ischemic attack; 6MWD, 6-minute walking distance.

**Supplementary Table 3. Baseline characteristics of ACO and non-ACO patients in the KOCOSS cohort**

|  | Non-ACO  (*n*=1,195) | ACO  (*n*=373) | *P*-value |
| --- | --- | --- | --- |
| Age, years | 69.3±7.6 | 68.2±7.7 | 0.020 |
| Sex, male | 1163 (96.9) | 364 (98.6) | 0.072 |
| Smoking status, current smoker | 350 (29.3) | 119 (32.3) | 0.261 |
| Smoking, pack-years | 44.3±24.6 | 46.5±25.0 | 0.140 |
| BMI (kg/m^2^) | 22.9±3.4 | 23.1±3.5 | 0.327 |
| Education (above high school) | 148 (12.5) | 62 (16.9) | 0.029 |
| Symptoms |  |  |  |
| Cough >3 months | 166 (33.0) | 46 (36.2) | 0.493 |
| Phlegm >3 months | 217 (39.6) | 71 (46.4) | 0.130 |
| mMRC | 1.33±0.87 | 1.34±0.93 | 0.892 |
| CAT score | 14.4±7.9 | 14.5±8.0 | 0.821 |
| SGRQ score | 32.0±21.1 | 33.4±22.1 | 0.274 |
| Blood eosinophil count | 139.2±75.7 | 531.0±374.9 | <0.001 |
| Past exacerbation | 230 (19.2) | 86 (23.3) | 0.083 |
| Past severe exacerbation | 123 (10.3) | 40 (1.8) | 0.755 |
| Comorbidities |  |  |  |
| Cardiovascular disease |  |  |  |
| Hypertension | 468 (39.0) | 149 (40.4) | 0.886 |
| Congestive heart failure | 36 (3.0) | 12 (3.3) | 0.805 |
| Ischemic heart disease | 54 (4.5) | 14 (3.8) | 0.841 |
| Peripheral vascular disease | 16 (1.3) | 7 (1.9) | 0.710 |
| Dyslipidemia | 145 (12.1) | 50 (13.6) | 0.553 |
| Diabetes mellitus | 227 (19.0) | 58 (15.8) | 0.164 |
| Cerebrovascular disease (TIA, stroke) | 8 (2.6) | 3 (2.9) | 0.864 |
| Gastroesophageal reflux | 139 (11.6) | 50 (13.6) | 0.564 |
| Past history of asthma | 421 (35.4) | 146 (39.9) | 0.117 |
| Spirometry |  |  |  |
| Post-BD FEV_1_, L | 1.72±0.59 | 1.75±0.58 | 0.521 |
| Post-BD FEV_1_, %predicted | 59.1±18.3 | 58.7±18.4 | 0.701 |
| Post-BD FVC, L | 3.39±0.77 | 3.40±0.78 | 0.803 |
| Post-BD FVC, %predicted | 82.3±16.1 | 81.2±16.4 | 0.268 |
| Post-BD FEV_1_/FVC | 50.6±12.1 | 51.2±11.9 | 0.356 |
| D_LCO_, % | 62.4±20.4 | 64.7±20.4 | 0.081 |
| BDR positivity | 119 (10.0) | 64 (17.2) | <0.001 |
| Exercise capacity, 6MWD (m) | 386.5±114.9 | 378.5±111.4 | 0.308 |
| Medication | 993 | 315 |  |
| ICS | 3 (0.3) | 0 (0) |  |
| Mono bronchodilator | 279 (28.1) | 98 (31.1) | 0.331 |
| Dual bronchodilator | 303 (30.5) | 79 (25.1) |  |
| ICS/LABA | 126 (12.7) | 44 (14.0) |  |
| Triple | 282 (28.4) | 94 (29.8) |  |
| Any ICS | 414 (41.4) | 138 (43.8) | 0.448 |

Data are presented as the mean±standard deviation or number (%).

AA, African American; ACO, asthma–COPD overlap; BMI, body mass index; CAT, COPD assessment test; COPD, chronic obstructive pulmonary disease; D_LCO_, diffusing capacity for carbon monoxide; FEV_1_, forced expiratory volume in 1 s; FVC, forced vital capacity; ICS, inhaled corticosteroid; LABA, long-acting β_2_ receptor agonist; mMRC, Modified Medical Research Council Dyspnea Scale; NHW, non-Hispanic white; SGRQ, St. George’s Respiratory Questionnaire; TIA, transient ischemic attack; 6MWD, 6-minute walking distance

**Supplementary Figure 1. Prevalence of ACO by race and ethnicity**

Values are presented as number, %.

AA, African American; ACO, asthma–COPD overlap; COPD, chronic obstructive pulmonary disease; NHW, non-Hispanic white.
