# Supplementary figures and images for: Racial Differences in Prevalence and Clinical Characteristics of Asthma–Chronic Obstructive Pulmonary Disease Overlap
Source: Front Med (Lausanne). 2021 Nov 22;8:780438. doi: 10.3389/fmed.2021.780438 (PMC8645561; doi:10.3389/fmed.2021.780438)

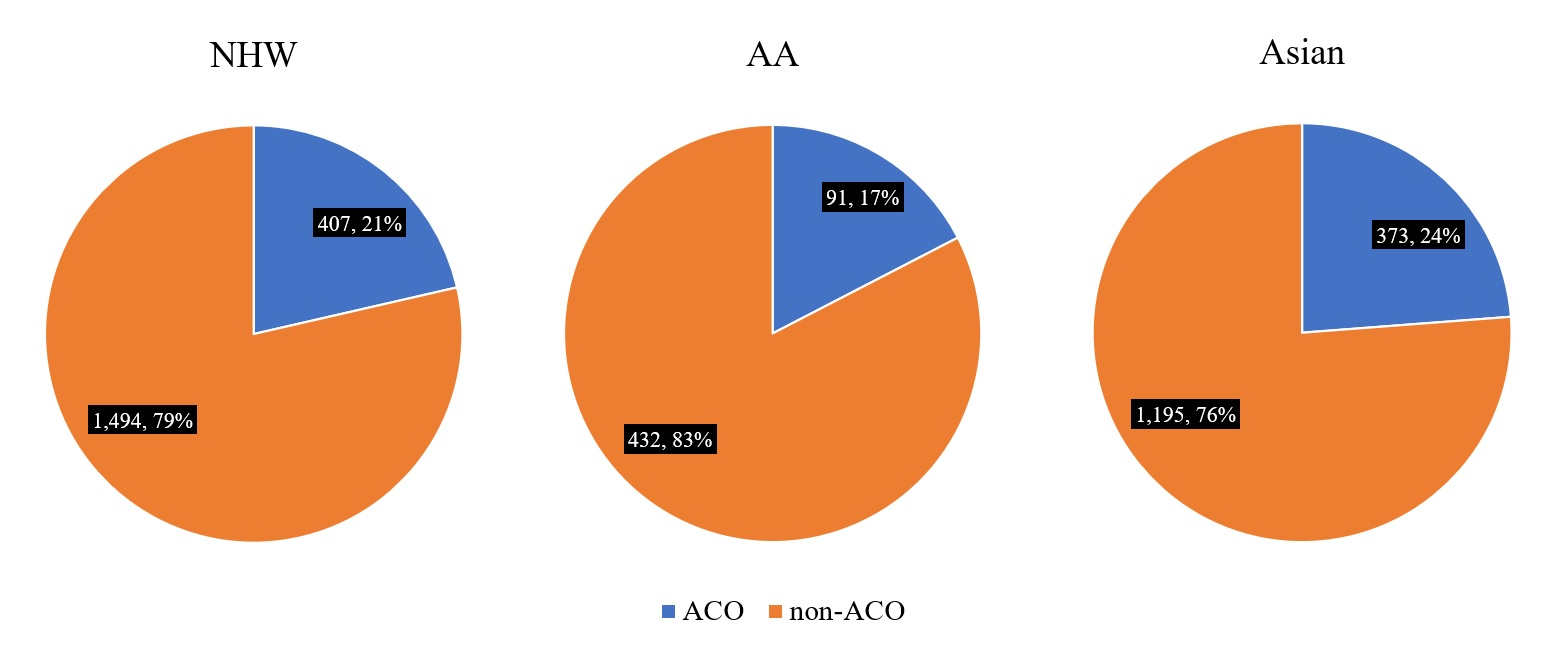

Supplement: Supplementary file 2 [file Image_1.TIFF]
